# Cribriform pattern does not have a significant impact in Gleason Score ≥7/ISUP Grade ≥2 prostate cancers submitted to radical prostatectomy

**DOI:** 10.1097/MD.0000000000022156

**Published:** 2020-09-18

**Authors:** Simone Flammia, Marco Frisenda, Martina Maggi, Fabio Massimo Magliocca, Antonio Ciardi, Valeria Panebianco, Ettore De Berardinis, Stefano Salciccia, Giovanni Battista Di Pierro, Alessandro Gentilucci, Francesco Del Giudice, Gian Maria Busetto, Michele Gallucci, Alessandro Sciarra

**Affiliations:** a Department of Maternal-Infant and Urological Sciences; b Department of Radiological, Oncological and Anatomopathological Sciences, Sapienza Rome University, Policlinico Umberto I, Rome, Italy.

**Keywords:** prostatic neoplasm, cribriform, radical prostatectomy

## Abstract

**Introduction:**

The aim of this study was to correlate cribriform pattern (CP) with other parameters in a large prospective series of Gleason score ≥7/ISUP grade ≥2 prostate cancer (PC) cases undergoing radical prostatectomy (RP).

**Methods:**

This is a prospective single-center study on 210 consecutive patients. Gleason pattern 4 and individual tumor growth patterns determination were performed either in biopsy or in surgical specimens for all patients.

**Results:**

At multiparametric magnetic resonance, a higher percentage of PI-RADS 5 was associated to CP (53.3% vs 17.7%, *P* = .038). CP was significantly and inversely (*r* = −0.261; *P* = .001) correlated with perineural invasion (PNI) but not with other pathological parameters. Kaplan-Meier analysis showed that mean biochemical (Bp) and radiological (Rp) progression-free survival were similar (Bp = *χ*^2^ 0.906; *P* = .341; Rp = *χ*^2^ 1.880; *P* = .170) independently to CP. In PNI positive cases, Bp-free survival was higher (*χ*^2^ = 3.617; *P* = .057) in cases without CP.

**Conclusions:**

In a homogeneous population excluding ISUP 1 cases, CP showed limited prognostic value. We first described an association with PNI and a prognostic value influenced by PNI status.

## 1 Introduction

The management of prostate cancer (PC) and clinical outcomes after treatments are significantly influenced by PC heterogeneity in histology. Clinical decision continues to depend upon serum prostate-specific antigen (PSA) levels, tumor stage, risk classes, and pathologic Gleason score (GS).^[1]^ Although patients with GS 6/ISUP grade 1 PC have excellent outcomes, those with GS ≥7/ISUP grade ≥2 can have variable results from therapies. Several biomarkers and characteristics identified on prostate biopsies (PB) have been suggested to provide valuable prognostic information that can have important therapeutic implications.^[2–6]^ Architectural heterogeneity of Gleason pattern 4 may be one of the explanations for the variable outcomes. Cribriform architecture is one of the 4 growth patterns recognized in Gleason pattern 4 (poorly formed, fused glands, glomerulation, and cribriform types) PC according to the ISUP 2014 modified Gleason system, and several works indicated it as an independent predictor of adverse clinical events and disease progression after radical prostatectomy (RP).^[7–13]^ Masoomiam et al^[14]^ analyzed the concordance between PB and RP diagnosis of cribriform PC in a prospective analysis on 245 cases. A cribriform pattern (CP) was reported in 22.9% of PB and in 42% of RP specimens, respectively. Higher percentages were reported by Hollemans et al^[15]^ on 186 PC with an invasive CP in 31% of PB and 69% of RP specimens, respectively. In a large cohort of pathological revised diagnostic PB on 1031 patients (European Randomized Study of Screening for Prostate Cancer), the incidence of CP accounted for 20%.^[8]^ In a multi-institutional study on 1275 patients submitted to RP, cribriform morphology was reported in 50% of cases.^[16]^

Even though the concept of CP may be associated with worsening of PC patient prognosis and outcomes, its significance and diagnostic relevance remain controversial and under the current grading system all the 4 distinct morphologies of Gleason pattern 4 are considered equal in terms of their impact on prognosis.^[17]^ Moreover, also its histological identification is not completely homogeneous in the different studies. Some authors evaluated the incidence of CP associated or not with an intraductal carcinoma as a distinct histomorphological entity,^[8,11]^ whereas others considered cribriform architecture together with the intraductal carcinoma because of their significant morphological overlap.^[14,15,18]^ Few studies proposed a classification of CP on the basis of a small or large extension in the specimens,^[11,19]^ whereas most of all^[12,13]^ considered the presence of any amount, even small foci of CP, associated with adverse outcomes.

These questions, and not univocal results from previous clinical studies, prompted us to correlate cribriform growth with other clinical and pathological parameters in terms of prognostic indicators in a large prospective series of GS ≥7/ISUP grade ≥2 PC cases submitted to RP.

In particular, the aim of the present study was: to correlate CP incidence at PB and at the following RP; to correlate CP incidence with either clinical parameters, such as PSA and multiparametric magnetic resonance (mpMRI) PI-RADS score, or recognized pathologic prognostic factors, such as GS, local stage, perineural invasion (PNI) and surgical margins (SM); to define the independent prognostic value of CP in terms of biochemical (Bp) and radiological progression (Rp).

## 2 Materials and methods

### 2.1 Population

In this prospective single-center study, patients with a histological diagnosis of GS ≥7/ISUP grade ≥2 PC considered for RP as primary therapeutic option in our Department were consecutively enclosed in the analysis. The protocol was approved by our internal ethical committee, and all patients gave their informed consensus for each procedure. Inclusion criteria were: histological diagnosis of prostatic adenocarcinoma, GS ≥7 (3 + 4), no distant metastases at clinical staging, determination of CP at pathologic specimens from PB and RP, RP as primary treatment option. Exclusion criteria were: absence of Gleason pattern 4 at pathology, concomitant active history or treatment for other neoplasms, androgen deprivation therapies, chemotherapies, pelvic radiation therapies or treatments with other agents that could influence prostate tumor growth.

From January 2016 to January 2019, 210 consecutive patients with GS ≥7/ISUP grade ≥2 PC submitted to RP in our Department corresponding to our inclusion and exclusion criteria were enclosed in our analysis.

### 2.2 Clinical parameters

All cases enclosed in the study were classified on the basis of clinical parameters described in Table 1. All cases underwent a standard random 14-cores biopsy of the prostate. Before surgery, clinical staging and risk category (D’Amico and EAU classification) assessment was homogeneously performed using total PSA determination and imaging (MRI, CT, bone scan) following European Association of Urology (EAU) guidelines.^[17]^ From 2017, patients underwent a mpMRI with Prostate Imaging Reporting and Data System (PI-RADS) score determination,^[20,21]^ performed by a single experienced radiologist (VP). In cases with PI-RADS score 3 to 5, additional targeted samples on the sites indicated by mpMRI were obtained. All patients underwent a laparoscopic or robot-assisted RP in our Department following EAU guidelines for indications.^[17]^ After surgery, all patients were followed at regular interval to determine time to Bp (confirmed total PSA progression >0.2 ng/mL), Rp (radiologically confirmed, local or distant), and overall survival, as recommended by the EAU guidelines.^[17]^

**Table 1:**
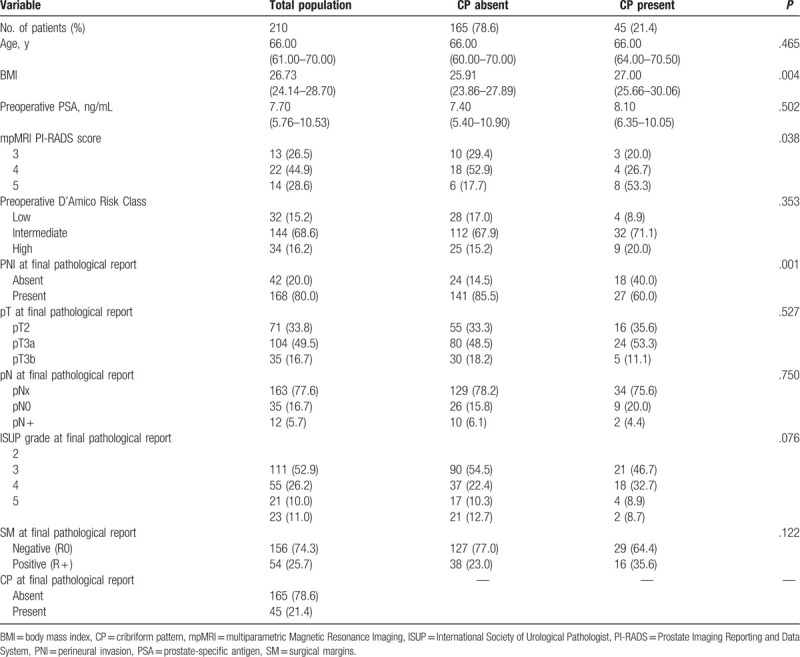
Patient characteristics. Number of cases (%); median (interquartie range). *P* value *χ*^2^ test or Fisher exact test or test *U* di Mann–Whitney.

### 2.3 Pathologic evaluation

All histological specimens from PB and RP were analyzed by our 2 dedicated uropathologists (FM and AC). GS and grade groups according to the World Health organization (WHO)/ISUP 2014 guidelines at PB and at surgery, pathologic staging using TNM classification, and SM status were routinely assessed in all cases. In particular, Gleason pattern 4 and individual tumor growth patterns determination were performed, either in PB or in surgical specimens for all patients.^[15]^ A cribriform architecture in Gleason pattern 4 was characterized by a solid proliferation with multiple punched-out lumina, without intervening stroma, and it was not distinguished from intraductal carcinoma through immunohistochemistry. We did not classify CP on the basis of its extension, yet we simply classified cases as positive or negative for CP presence.

### 2.4 Statistical analysis

For statistical evaluation SPSS Statistics program was used. Descriptive statistical methods, such as number of cases, mean ± standard deviation (SD), median and interquartile range (IQR) were used. For the comparison of quantitative data and pairwise intergroup comparisons of variables Mann Whitney test was performed. For comparison of qualitative data, Fisher exact test and *χ*^2^ test were used. Pearson correlation analysis was also performed. Univariate and multivariate Cox proportional analyses considering clinical and pathological parameters were used. Kaplan-Meier survival curves related to CP and to the clinical outcomes were obtained. Statistical significance was set at *P* < .05.

## 3 Results

Baseline characteristics of the 210 cases included in our analysis are described in Table 1. The median follow-up time after surgery was 22 months (IQR 12–36). At PB, a CP was found in 15.2%, whereas at surgery in 21.4% of cases.

### 3.1 Correlation among CP incidence and clinical parameters

Clinical parameters, such as age and preoperative PSA were not significantly different between cases with and without CP (*P* > .50) (Table 1). Similarly, the distribution of PC risk classes did not significantly differ regarding cribriform status (*P* = .353). Pearson correlation analysis showed a not significant correlation between cribriform status and risk classes (r = 0.095; *P* = .172) (Table 2). At mpMRI, the distribution of PI-RADS 3 score was similar between the 2 groups, whereas a higher percentage of PI-RADS 5 score was found in cases with CP (53.3% vs 17.7%; *r* = 0.598; *P* = .019) (Tables 1 and 2).

**Table 2:**
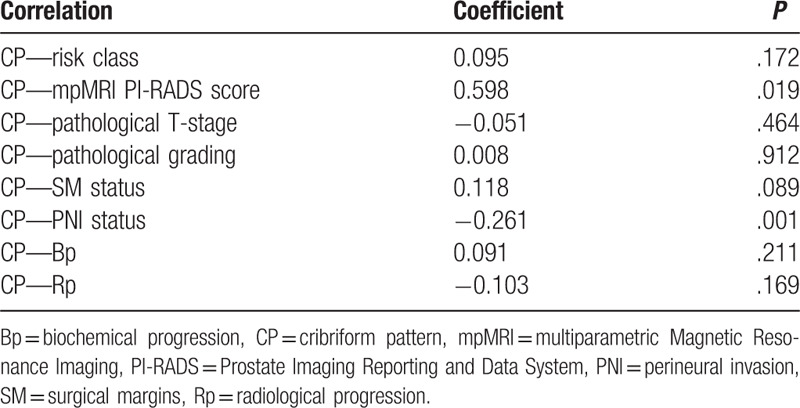
Correlation coefficients among Cribriform status and other clinical and pathological parameters. Spearman coefficient.

### 3.2 Correlation among CP incidence and pathological parameters

**Figure 1:**
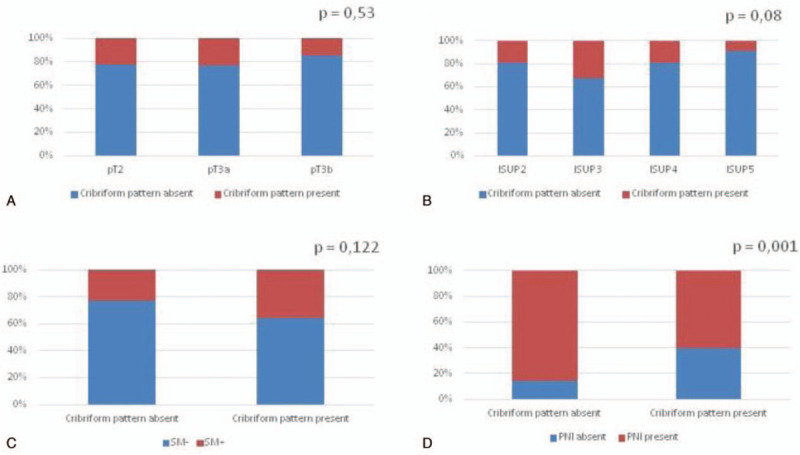
Bar-chart showing the percentage of patients with cribriform pattern (absent, present) at surgery according to: (A) pathological stage (pT2, pT3a, pT3b); (B) ISUP grading (2, 3, 4-5); (C) surgical margins (SM); (D) perineural invasion (PNI) status. *χ*^2^ analysis.

CP was significantly and inversely correlated with PNI (*r* = −0.261; *P* = .001), but not with other pathological parameters, such as local stage (*r* = −0.019; *P* = 0.789), grading (*r* = 0.008; *P* = .912) and SM (*r* = 0.118; *P* = .089) (Table 2). In particular, the incidence of PNI was significantly higher in the group without CP, when compared to the CP positive group (85.5% vs 60.0%, respectively; *P* = .001), whereas the percentage of extracapsular PC at surgery was similar between the 2 groups (66.7% and 64.4% in CP negative and positive cases, respectively) (Table 1; Fig. 1A and D). The distribution of ISUP grading was similar between the 2 groups (*P* = .076), although the presence of CP increased from ISUP 2 to ISUP 3 groups (18.9% vs 32.7%, respectively), and SM positivity was slightly higher in the group with CP than in cases without (35.6% versus 23.0%, respectively; *P* = .122) (Table 1, Fig. 1B and C).

### 3.3 CP and postoperative survival results

**Figure 2:**
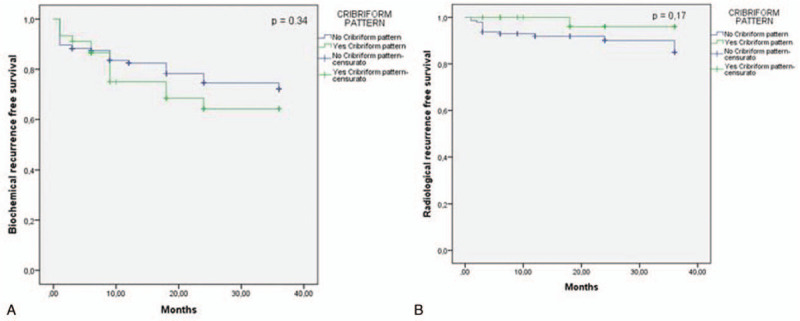
Kaplan-Meier curves showing biochemical (A) and radiological (B) progression-free survival following radical prostatectomy according to cribriform pattern (Biochemical progression-free survival log Rank Mantel—Cox: *χ*^2^ 0.906; *P* = .341; Radiological progression-free survival log Rank Mantel—Cox: *χ*^2^ 1.880; *P* = .170).

During the postoperative follow-up, 21.1% of our population developed a Bp, and 8.3% a Rp; all patients are alive. CP status was not significantly correlated with Bp and Rp development (*r* = 0.091; *P* = .211 and *r* = −0.103; *P* = .169, respectively) (Table 2). Kaplan-Meier survival analysis showed that mean Bp-free survival was similar between the 2 groups (29.16 ± 1.17 and 26.76 ± 2.14 months in CP absent and present groups, respectively; *χ*^2^ 0.906; *P* = .341) (Fig. 2 and Table 3). Similarly, Rp-free survival did not significantly differ between CP present and absent cases (33.18 ± 0.81 and 35.28 ± 0.71 months, respectively; *χ*^2^ = 1.880; *P* = .170) (Fig. 2 and Table 3).

**Table 3:**
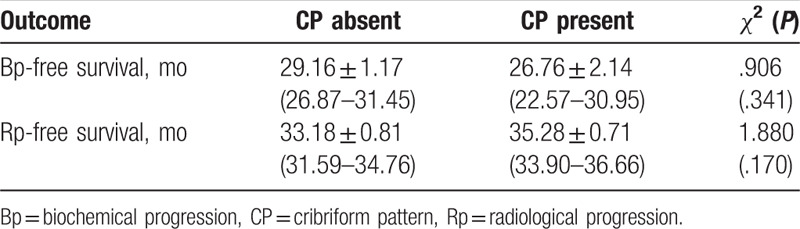
Clinical outcomes at follow-up after surgery according to Cribriform results at surgery (mean ± standard error and 95% CI). Log Rank Mantel Cox of *χ*^2^.

**Figure 3:**
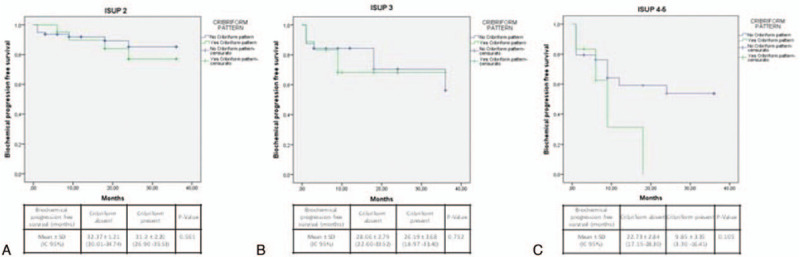
Kaplan-Meier curve showing biochemical progression-free survival following radical prostatectomy according to ISUP grading and cribriform pattern. (A) ISUP 2 (log Rank Mantel—Cox: *χ*^2^ 0.339; *P* = .561), (B) ISUP 3 (log Rank Mantel—Cox: *χ*^2^ 0.100; *P* = 0.752) and (C) ISUP 4-5 (log Rank Mantel—Cox: *χ*^2^ 2.621; *P* = .105).

**Figure 4:**
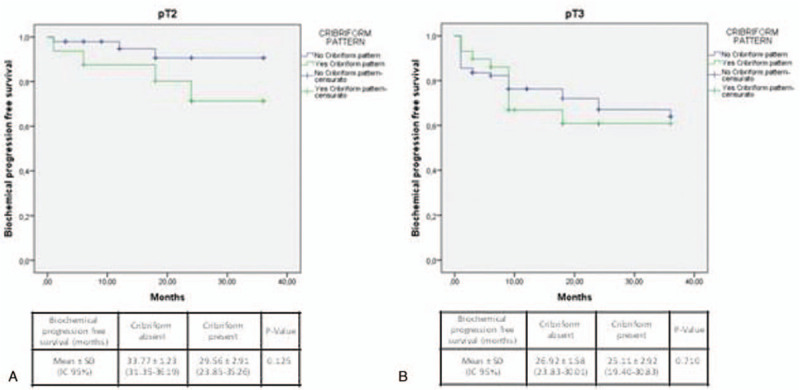
Kaplan-Meier curve showing biochemical progression-free survival following radical prostatectomy according to pathologic T-stage and cribriform pattern. (A) pT2 (log Rank Mantel—Cox: *χ*^2^ 2.356; *P* = .125) and (B) pT3 (log Rank Mantel—Cox: *χ*^2^ 0.139; *P* = 0.710).

**Figure 5:**
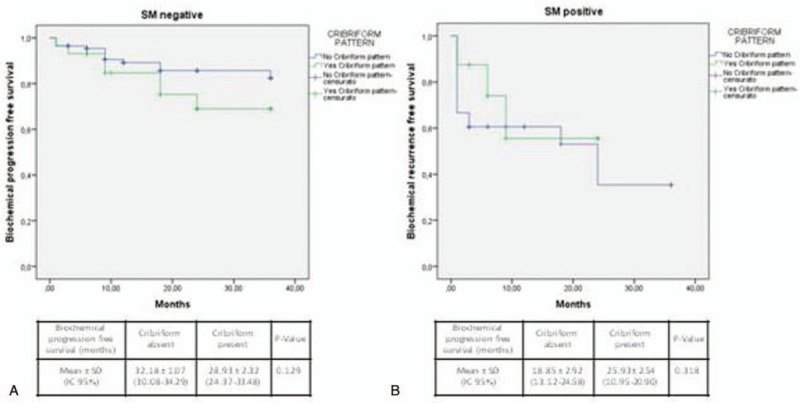
Kaplan-Meier curve showing biochemical progression-free survival following radical prostatectomy according to surgical margins (SM) status and cribriform pattern. (**A**) SM positive (log Rank Mantel—Cox: *χ*^2^ 0.998; *P* = .318) and (**B**) SM negative (log Rank Mantel—Cox: *χ*^2^ 1.980; *P* = .159).

**Figure 6:**
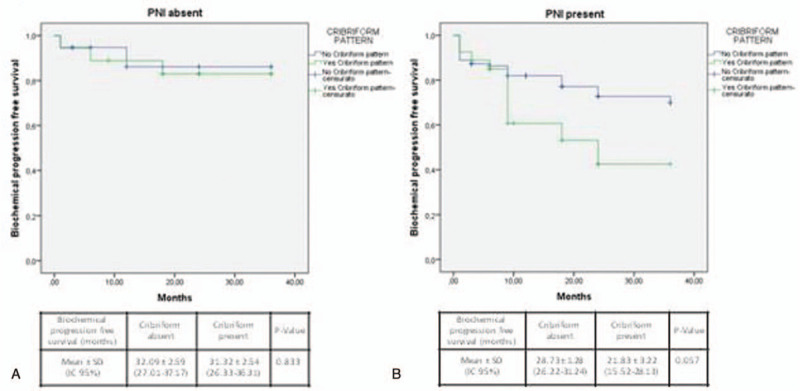
Kaplan-Meier curve showing biochemical progression-free survival following radical prostatectomy according to perineural invasion (PNI) status and cribriform pattern. (A) PNI positive (log Rank Mantel—Cox: *χ*^2^ 3.617; *P* = .057) (B) PNI negative cases (log Rank Mantel—Cox: *χ*^2^ 0.044; *P* = .833).

Stratifying cases by ISUP grading (Fig. 3), T staging (Fig. 4), or SM status (Fig. 5), Kaplan-Meier analysis showed no significant differences in terms of Bp-free survival according to CP status in each ISUP grading or T staging group. However, in ISUP 4-5 cases the Bp-free survival rate, although not statistically significant, was higher in the group without CP, when compared to the group with CP (22.73 ± 2.8 and 9.85 ± 3.3 months, respectively). Stratifying our population by PNI status (Fig. 6), in the PNI-positive group Bp-free survival rate was higher in cases without CP, when compared to cases with CP (28.73 ± 1.28 and 21.83 ± 3.22 months; *χ*^2^ = 3.617; *P* = .057).

### 3.4 Cox regression analysis for CP prognostic value

In Table 4, Cox regression analysis was carried out to identify predictors of Bp in our population. Univariate analysis showed that CP was not a significant predictor of Bp, with a 1.36 fold higher risk, when compared with cases without CP (hazard ratio [HR] = 1.36, 95% confidence interval [CI] 0.71–2.62; *P* = .360) (Table 4). On the contrary, significant predictors of Bp at univariate analysis resulted pathological stage, risk classes (limited to high risk class), ISUP grading, and SM status (*P* < .05) (Table 4).

**Table 4:**
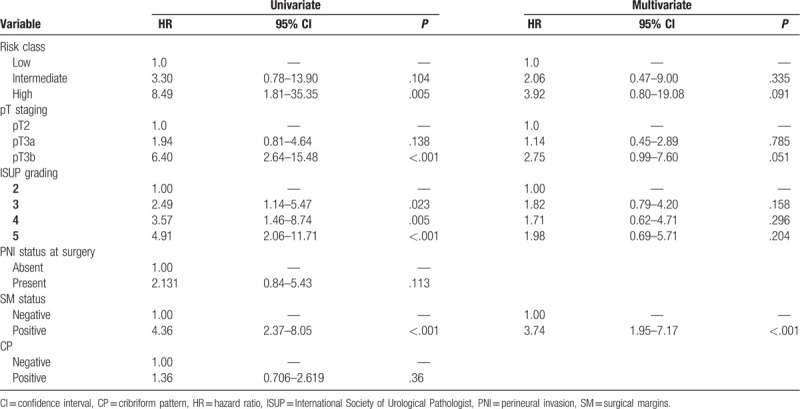
Cox regression analysis for the identification of the pathological predictors for biochemical progression after surgery.

## 4 Discussion

CP in PC is suggested as a possible indicator of more aggressive disease able to negatively influence free survival rates after surgery.^[8,9]^ However, in International guidelines under the current grading system, all the 4 distinct morphologies of Gleason pattern 4 are considered equal in terms of their impact on prognosis.^[17]^ Some authors distinguished the incidence of cribriform architecture associated or not with an intraductal carcinoma,^[11]^ whereas others^[15]^ considered them independently because of their significant morphological overlap. Few studies proposed a classification of CP on the basis of a small or large extension in the specimens.^[11,19]^

In our prospective analysis on a large series of PC cases submitted to RP, we decided to limit the evaluation to ISUP grade 2-5 cases. Including also well differentiated GS 6/ISUP grade 1 cases may produce more significant results, yet less focused on the prognostic role of cribriform architecture in the presence of Gleason pattern 4 tumor. In our population, the incidence of CP at biopsy (15.2%) and at surgery (21.4%) was lower than in previous studies (biopsy 20%–31%; surgery 42%–69%).^[14,15,18]^ Of note, although with different rates, the concordance of biopsy and corresponding RP diagnosis of this pattern remains similar and limited. Massomiam et al^[14]^ on 245 PC cases submitted to RP showed a prevalence of CP of 22.9% at biopsy, and 42.0% at surgery, with a 44.7% sensitivity of biopsy diagnosis. Ericson et al^[18]^ on 216 PC cases, showed a 47.5% of biopsies positive for cribriform architecture, with a 56.5% sensitivity for biopsy results when compared to RP. The conclusion was that biopsy is not enough sensitive for detecting the real cribriform morphology incidence in a PC population considered for primary treatment, and this point should be more relevant whether the population is selected for not surgical procedures.

mpMRI is now considered a recommended imaging to direct PB.^[21]^ Gao et al^[22]^ on 215 PC cases submitted to RP showed a higher percentage of PI-RADS 5 and lower of PI-RADS 4 in cribriform-positive (71.8% and 24.6%, respectively) than in cribriform-negative (25.7% and 56.2%, respectively) (*P* < .001) cases. Similarly, in our population, CP status was associated with a higher percentage of PI-RADS score 5 (53.3% vs 17.7%) (*P* = .038).

Different studies suggested a positive association between CP and tumor stage, grading and worse progression-free survival. Haffner et al^[13]^ on 367 cases submitted to RP, mainly ISUP 2-5, showed that cribriform morphology was significantly associated with an increased extent in Gleason pattern 4 (*r* = 0.277; *P* < .014), higher Gleason grade (*r* = 0.235; *P* < .001) and stage (*r* = 0.17; *P* < .003), but not SM status. No results in terms of survival were described. Sarbay et al^[12]^ on a population of 185 RP cases including a high percentage of GS 6, showed a significantly higher percentage of CP in GS ≥7 (3 + 4) versus GS 6 (3 + 3) (*P* < .001), but not significantly (*P* = 0.60) higher in GS 8 when compared to GS 7. Similarly, the incidence of extracapsular extension in GS 7 was not significantly different (*P* = 0.331) regarding cribriform status. Of note, their analysis was carried out assessing CP associated with Gleason patterns 3, 4, and 5; therefore, positive results of this study were strongly conditioned by the inclusion of Gleason pattern 3 in their evaluations. Hollemans et al^[11]^ restricted their evaluation on 420 ISUP grade 2 PC submitted to RP, distinguishing between small and large cribriform pattern. The majority of cases (85.1%) showed a small pattern, whereas only the few (14.9%) cases with a large CP showed significantly (*P* < .001) higher rate of extracapsular disease.

In our population, considering only ISUP grade 2-5 PC, we did not find a significant correlation between CP and neither T stage nor ISUP grading. The percentage of extracapsular disease was very similar (66.7% and 64.4%) regarding cribriform status, the presence of CP increased from ISUP2 (18.9%) to ISUP 3 (32.7%) groups, and SM positivity was slightly higher in the group with CP (35.6%) than in (23.0%) cases without (*P* = .122). In particular, PNI and CP seemed to be inversely correlated and the incidence of PNI was significantly (*P* = .001) higher (85.5%) in the group without CP than in (60.0%) the cribriform positive group.

Considering only ISUP 2-5 cases, in our population, CP status was not able to significantly influence Bp and Rp development (HR 1.36; 95% CI 0.70–2.61; *P* = .36). Trudel et al,^[9]^ on a population of 246 RP including 51.6% of ISUP 1 PC, reported a significant negative prognostic effect on Bp-free survival, although HR was only 1.03 (95% CI 1.01–1.04). Kweldam et al^[8]^ using the European Randomized Study of Screening for Prostate Cancer trial, analyzed a large population of 1031 PC cases including 47% of ISUP 1 tumors and different primary treatments (RP, radiotherapy, and watchful waiting). In this large heterogeneous population, the presence of CP was analyzed at biopsy level and it was significantly associated with worse disease-specific survival (HR 6.3, 95% CI 3.9–10; *P* < 0.001). A specific evaluation of Bp- or Rp-free survival was not presented in the publication. Stratifying the results on the basis of ISUP grading, differences reached statistical significance only in ISUP 2, 4, and 5 (*P* < .001).

In our experience, stratifying cases on the basis of ISUP grading or T staging, Kaplan-Meier analysis showed no significant differences in terms of Bp-free survival according to CP status. However, in ISUP 4-5 cases the Bp-free survival, although not statistically significant, was higher in the group without (22.73 ± 2.8 months) when compared to the group with CP (9.85 ± 3.3 months). In the PNI-positive group, Bp-free survival was significantly (*χ*^2^ = 3.617; *P* = .057) higher in cases without (28.73 ± 1.28 months) when compared to cases with CP (21.83 ± 3.22 months).

Limitation associated to our study is the postoperative follow-up that does not consent to have results in terms of disease specific or overall survival.

## 5 Conclusions

Some considerations can be obtained. In the literature, positive results regarding the prognostic value of CP evaluation in PC could be influenced by the heterogeneity of the population. The usefulness of a classification in large and small CP extension seems to be not justified since only few studies reported, and most of cases (85%) had small extension. Considering this parameter in a homogeneous population submitted to RP and excluding ISUP 1 cases its association with worse pathological features in terms of grading and T staging is limited and its prognostic value in terms of progression-free survival results significant only after stratifications on the basis of other prognostic indicators. We first described an inverse association between CP and PNI and a prognostic value influenced by PNI status.

## Author contributions

**Conception and design:** Alessandro Sciarra, Martina Maggi, Simone Flammia.

**Administrative support:** Marco Frisenda, Giovanni Battista Di Pierro, Gian Maria Busetto.

**Provision of study materials or patients:** Valeria Panebianco, Michele Gallucci, Ettore De Berardinis, Stefano Salciccia.

**Collection and assembly of data:** Simone Flammia, Francesco Del Giudice, Alessandro Gentilucci.

**Data analysis and interpretation:** Martina Maggi, Antonio Ciardi, Fabio Massimo Magliocca.

**Manuscript writing:** Simone Flammia, Marco Frisenda, Martina Maggi, Fabio Massimo Magliocca, Antonio Ciardi, Valeria Panebianco, Ettore De Berardinis, Stefano Salciccia, Giovanni Battista Di Pierro, Alessandro Gentilucci, Francesco Del Giudice, Gian Maria Busetto, Michele Gallucci, Alessandro Sciarra.

**Final approval of manuscript:** Simone Flammia, Marco Frisenda, Martina Maggi, Fabio Massimo Magliocca, Antonio Ciardi, Valeria Panebianco, Ettore De Berardinis, Stefano Salciccia, Giovanni Battista Di Pierro, Alessandro Gentilucci, Francesco Del Giudice, Gian Maria Busetto, Michele Gallucci, Alessandro Sciarra.

## Abbreviations

Abbreviations: Bp = biochemical progression, CP = cribriform pattern, CT = computer tomography, EAU = European Association of Urology, GS = Gleason score, mpMRI = multiparametric Magnetic Resonance Imaging, PB = prostate biopsy, PC = prostate cancer, PI-RADS = Prostate Imaging Reporting and Data System, PNI = perineural invasion, PSA = prostate-specific antigen, Rp = radiological progression, RP = radical prostatectomy, SM = surgical margins.
